# Wearable and Flexible Ozone Generating System for Treatment of Infected Dermal Wounds

**DOI:** 10.3389/fbioe.2020.00458

**Published:** 2020-05-19

**Authors:** Alexander Roth, Ahmed Elkashif, Vidhya Selvamani, Rachel Abigail Stucky, Mohamed N. Seleem, Babak Ziaie, Rahim Rahimi

**Affiliations:** ^1^Birck Nanotechnology Center, Purdue University, West Lafayette, IN, United States; ^2^School of Mechanical Engineering, Purdue University, West Lafayette, IN, United States; ^3^Department of Comparative Pathobiology, College of Veterinary Medicine, Purdue University, West Lafayette, IN, United States; ^4^School of Materials Engineering, Purdue University, West Lafayette, IN, United States; ^5^Department of Basic Medical Sciences, Purdue University, West Lafayette, IN, United States; ^6^School of Electrical and Computer Engineering, Purdue University, West Lafayette, IN, United States

**Keywords:** ozone, antibiotic resistance, wound therapy, smart patch, dermal wound

## Abstract

Wound-associated infections are a significant and rising health concern throughout the world owing to aging population, prevalence of diabetes, and obesity. In addition, the rapid increase of life-threatening antibiotic resistant infections has resulted in challenging wound complications with limited choices of effective therapeutics. Recently, topical ozone therapy has shown to be a promising alternative approach for treatment of non-healing and infected wounds by providing strong antibacterial properties while stimulating the local tissue repair and regeneration. However, utilization of ozone as a treatment for infected wounds has been challenging thus far due to the need for large equipment usable only in contained, clinical settings. This work reports on the development of a portable topical ozone therapy system comprised of a flexible and disposable semipermeable dressing connected to a portable and reusable ozone-generating unit via a flexible tube. The dressing consists of a multilayered structure with gradient porosities to achieve uniform ozone distribution. The effective bactericidal properties of the ozone delivery platform were confirmed with two of the most commonly pathogenic bacteria found in wound infections, *Pseudomonas aeruginosa* and *Staphylococcus epidermidis*. Furthermore, cytotoxicity tests with human fibroblasts cells indicated no adverse effects on human cells.

## Introduction

Skin and soft tissue infections (SSTIs) are a major health and financial burden for millions of people worldwide. In 2016, SSTIs comprised 3.5% of all emergency room visits (Niska et al., 2016). Furthermore, the average cost of a hospital visit resulting from an SSTI is about $8,000 (SSTI, 2018). These numbers are only expected to rise in the years to come due to the aging population and the increasing prevalence of diabetes associate non-healing wounds and bedsores. To complicate the issue even further, many of these infections can be caused by bacteria that are resistant to common forms of treatment. Infections caused by drug-resistance bacteria have become a significant problem and now affecting over 2 million people in the US each year (Centers for Disease Control and Prevention, 2018). For instance, methicillin-resistant *Staphylococcus aurous* (MRSA), has been noted to kill more Americans every year than HIV/AIDS, emphysema, or homicide (Federal Bureau of Investigation, 2017; CDC, 2018; Kourtis et al., 2019; Lei et al., 2019). This alarming decrease in antibiotic efficacy has been brought on by a number of factors, but a primary culprit is the commonality of antibiotics usage in society today, especially for inappropriate or unnecessary indications (Ventola, 2015). Simultaneously, major drug companies have reduced the number of antibiotics they are developing. This is mainly due to a significantly reduced return on investment for antibiotic research and development compared to the drugs for chronic conditions such as diabetes, heart disease, and cancer (Ventola, 2015).

Recently, there has been increased effort toward the development of alternative (non-antibiotic) materials and treatments for bacterial infections (Kowalski et al., 1998; Laroussi et al., 2000; Fridman et al., 2005; Fontes et al., 2012; Guan et al., 2013; Korshed et al., 2016). For example, metallic nanoparticles of noble metals such as silver have shown to exhibit antimicrobial properties for a wide range of bacteria and utilized in various advanced wound dressings (Maneerung et al., 2008; Rujitanaroj et al., 2008). Despite having effective antimicrobial activity, many studies have also shown that silver nanoparticles are cytotoxic, and cause damage to cellular components such as DNA and cellular membrane (Korshed et al., 2016). Other materials include polyvinyl-pyrrolidone, a non-ionic synthetic polymer, which allows for gradual release of free iodine with antimicrobial effects. Yet, povidone-iodine and its different complex forms have also been shown to delay wound healing by inhibiting fibroblast aggregation and leukocyte migration (Álvarez-Paino et al., 2017). Free radical and ionized gasses generated by cold atmospheric plasma (CAP) have also shown to be an effective alternative therapeutic tools, providing both antimicrobial properties and help promoting wound healing, and tissue regeneration (through activation of growth factors and stimulation of angiogenesis) (Laroussi et al., 2000; Fridman et al., 2005). Although a promising approach, only a few devices and systems using CAP for wound treatment have been adopted by the patients or their caregivers. One major impediment of their widespread adoption is the system cost and complexity. These devices (e.g., Microplaster from Adtech Ltd) use plasma gun/torch that require high voltages, carrier gas (typically argon) and need to be operated by trained personnel in an outpatient setting. Similarly, smart wound dressings have been developed to help increase wound healing through delivery and sensing of factors such as oxygen, as well as drug delivery and providing optimal wound healing conditions (Gupta et al., 2010; Mostafalu et al., 2015; Ziaie et al., 2018).

Ozone therapy is another gas phase antimicrobial therapeutic modality. Ozone is known to inactivate bacteria, viruses, fungi, yeast, and protozoa through the oxidation of phospholipids and lipoproteins in the cell envelope, which leads to weakened or destroyed bacterial walls (Elvis and Ekta, 2015). Ozone has been used for many years to deodorize and sanitize hard to reach surfaces and disinfect drinking water (Glaze, 1987). More recently, ozone therapy has been suggested for dental plaque removal and caries repair, although its efficacy in teeth re-mineralization is still debatable (Saini, 2011). As related to wound therapy, several groups have proposed the use of ozone to treat antibiotic resistant skin infections. In their *in vitro* validation experiments, ozone is applied at high concentrations, often in excess of 1000 ppm to deactivate cultured bacteria colonies over a short time period. Fontes *et al.* showed that at 9500 ppm, multiple strains of bacteria were inhibited after just 5 min of ozone application (Fontes et al., 2012). Kowalski et al. (1998) were able to achieve at least 95% reduction in *Escherichia coli* and *Staphylococcus aureus* using treatments of 600 and 1500 ppm, respectively, in under a minute. In addition to its antimicrobial properties, ozone also stimulates wound healing through applied oxidative stress, which leads to increased production and migration of wound healing factors, and increased oxygen levels at the wound site (Filippi, 2001; Sen et al., 2002; Martínez-Sánchez et al., 2005; Lim et al., 2006; Valacchi et al., 2011; Wainstein et al., 2011; Zhang et al., 2014; Degli Agosti et al., 2016).

**FIGURE 1:**
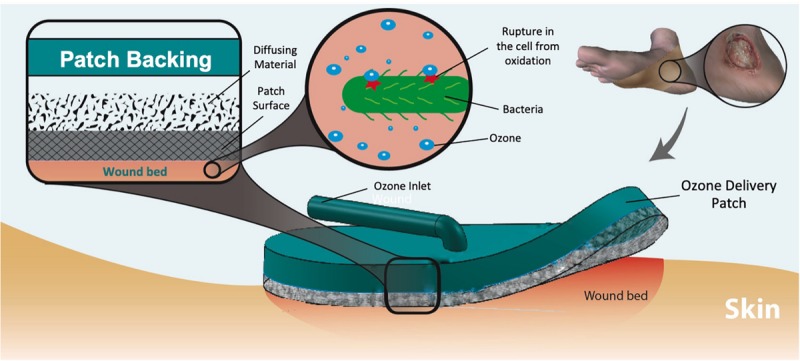
Dermal wound with bacteria and applied ozone treatment patch. The ozone delivered to the wound eliminates the microbes through oxidation of the cell wall. Multiple layers of material create a pore size “gradient” to increase the uniformity of output flow.

Ozone therapy offers a unique advantage as related to its relative ease of generation and controllability. Ozone can be artificially produced (using high voltages or UV-light) within small portable units carried by the patient, allowing for low-dose continuous or long-term treatment regimen, reducing the adverse effects of high concentrations on normal skin cells (Fontes et al., 2012). A wearable ozone generation platform would allow for ozone treatment to be administered topically to a patient outside of a clinical setting. In this work, we demonstrate the design, fabrication, and test of a portable and wearable platform capable of local and on demand administration of ozone to the wound site (Figure 1). The platform is composed of a portable ozone-generating unit connected to a flexible and porous gas permeable patch, which delivers the generated ozone to the wound site. For effective ozone treatment, the patch requires uniform permeation of gas through the dressing without significant resistance. Additionally, the material in contact with the wound must exhibit hydrophobic properties to allow for contact with biofluids on the wound surface without blocking the exposed pores. Another important design feature is an internal dispersion layer used to improve uniformity of the output flow. The varying pore size between the dispersion layer and patch material create a “gradient” of pore sizes, which help to distribute the flow of gas from a single input point across the patch surface.

**FIGURE 2:**
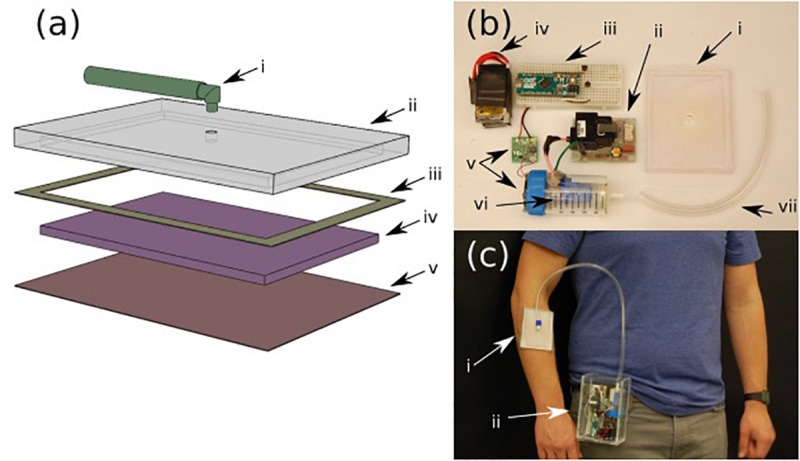
**(a)** Various layers of the ozone treatment patch (i) input tubing, (ii) PDMS backing, (iii) 3M binding tape, (iv) intermediate dispersion layer, and (v) PDMS treated fabric. **(b)** Components of the portable ozone treatment system: (i) assembled patch, (ii) ozone generator, (iii) microcontroller with control circuit, (iv) rechargeable battery pack, (v) micro-blower with driver circuit, (vi) ozone generation chamber, and (vii) connection tubing. **(c)** Portable system (ii) attached to the belt with the patch on the forearm (i).

Figure 2a shows an expanded view schematic of the dressing. The patch was designed to be low cost and disposable. A cost analysis of the patch has been included in the Supplementary Data Sheet 1. The backing of the patch (Figure 2ai) is made from PDMS. The patch (Figure 2aiv) itself is a synthetic Rayon-Spandex knit fabric. This material was chosen for its high gas permeability and low cost. To introduce hydrophobicity on the wound touching surface, the patch was coated in a diluted PDMS solution. Inside the patch, an intermediate flow dispersion layer (Figure 2aiii) was added to help generate a more uniform output flow. This layer was made of a low-cost woven polymer (polyester batting) with a greater porosity for the gas to pass through, and is inserted inside the cavity of the PDMS backing. By introducing this intermediate layer, we were able to create a gradient of pore sizes, which adds a small resistance to flow and causes the gas to distribute more further toward the extremities of the patch, and leads to a more consistent application of ozone across the wound. The patch was sealed to the backing with a bonding double-sided tape (Figure 2aii).

Both the portable ozone generation system and the patch were fabricated using rapid prototype techniques that can be simply adapted to standard scalable manufacturing technologies used in production of wound dressings. The ozone generation system consists of a small, low voltage commercial ozone generation system (Figure 2bii) and a micro-blower fan (Figure 2bv). The ozone generator leads were enclosed in a sealed chamber (Figure 2bvi) with an outlet connected to the patch (Figure 2bi). The micro-blower was mounted at one end of the sealed chamber such that, when running, it would create a constant flow within the chamber, blowing ozonized air at a constant rate through the outlet into the patch (Figure 2bi). Both the ozone generator and micro-blower included driver boards (Figure 2bii,v), which were assembled in a portable box along with a battery pack (Figure 2biv) and control circuit (Figure 2biii). The assembled device can be easily worn (as shown in Figure 2c) with the housing containing all of the necessary generation components (ii) and the patch attached to the patient with a medical adhesive such as Tagaderm (i).

## Materials and Methods

### Ozone Generating System

In order to generate ozone from the ambient air, the system utilizes a small commercial ozone generator, Murata model MHM500. Murata MZB1001T02 piezoelectric micro-blower and manufacturer’s driver circuit was used to push the generated ozone through the tubing and dressing. These components, along with a battery and microcontroller circuit were arranged inside a 135 mm × 90 mm × 70 mm acrylic housing.

### Patch Fabrication

The patch was fabricated from a 95% Rayon 15% Spandex knit fabric. The PDMS coating was applied by first mixing the PDMS elastomers and curing agent at a 1:10 weight ratio, and then diluting with heptane at a volume ratio of 1:5. Pretreated patches were exposed to plasma treatment over the entire surface in order to increase the bonding of the PDMS with the patch. The patches were then submerged in the diluted PDMS solution and set in a 70°C oven to dry for approximately 1 h. The flexible patch backing was also fabricated from PDMS. The same 1:10 ratio of elastomers to curing agent were mixed and poured into a mold created for the backing. The backing is shaped to match the needed patch, with a hole in the top for the addition of a connection point for the gas tubing. The mold was then left in a vacuum chamber for 1 h before being placed in the oven to cure before being removed. Before attaching the patch, a piece of low-density fiber mesh (1/8 in thick polyester batting), used as the intermediate flow dispersion layer, was cut to size and added. To attach the backing to the patch, 3M 300LSE double-sided tape was applied between the backing and the patch after each surface was plasma treated to increase bond strength. Once the patch and backing were taped together, they were again put in the oven to cure. Once bounded, the connection with the ozone generating system was made via a 5 mm inner diameter tubing.

### Patch Characterization

Hydrophobicity tests utilized a Ramé-Hart Model 290 F1 Advanced Goniometer to measure contact angle (CA) between water droplets and the patch material. The mechanical loading cycles were applied in compression to the patch using a ADMET eXpert 4000 connected to an MTESTQuattro in 25 cycle increments, with a bending radius of 13.5 mm. Measurements were taken in triplicate. The microscope images were captured through a Bausch & Lomb MicroZoom II microscope with a 2.25× magnification lens, and the SEM imaging was done using a S-Hitachi FE SEM. Pore size measurements were made through image analysis on ImageJ software using the measurement tool scaled to the given image. To experimentally determine the flow resistance, an Omega DPG 4000 pressure sensor was connected in series with the patch, and pressure readings were obtained for various flow rates as generated by a New Era 1000 syringe pump. The test was performed on the patch material before and after PDMS coating, with and without the intermediate dispersion layer, and on Tyvek. When characterizing the ozone concentration output, a voltage input of 12V DC was delivered to the ozone generator, and the remote input was grounded. The ozone concentration was measured with a D16 Protasense III gas sensor with the 0–1000 ppm ozone module (0–1358) set to the 0–200 ppm. Thirteen data points collected for each setting. To test how the ozone distribution changed over time, ozone detection strips were created by soaking the same Rayon-Spandex fabric samples cut in the same shape as the patch in potassium iodide solution (235 μM). The detection strip was then dried in a 70°C oven for approximately 1 h. The detection strips were then adhered to a 110 mm by 95 mm 1/8 in acrylic sheet to mimic a flat application area for testing. Tests were conducted three times each with the patch facing directly up, and the test strips placed on top to cover the patch area. Pictures were taken of the test strip every 10 s for 2.5 min while the device was ran at 12 V DC input for the ozone generator, and 8 V DC input into the micro-blower. Tests were conducted for the patch both with and without the intermediate flow dispersion layer. Additionally, Using the Portasense III gas sensor, ozone concentration readings were measured at each point on the patch as indicated in Figure 7a. The concentration mapping experiment used the same inputs of 12 V DC for the ozone generator and 8 V DC input for the micro-blower. In each case above, average values and standard deviation were calculated from the datasets collected.

### Antimicrobial Tests

The clinical isolates of *Pseudomonas aeruginosa* and *Staphylococcus epidermidis* used in this study were obtained from Biodefense and Emerging Infections Research Resources Repository (BEI Resources) and American Type Culture Collection (ATCC). All the media used in this study were purchased from chemical vendors: dextrose, trypticase soya agar (TSA), and trypticase soya broth (TSB) were purchased from Becton, Dickinson, and Company (Cockeysville, MD, United States).

To test the susceptibility of the bacterial strains to ozone treatment, an overnight culture of each strain was diluted 1:100 in TSB and incubated for 3 h till OD_600_ = 1 to establish a mid-log phase culture. The resulting inoculum was diluted 1:1000 in M9 Buffer supplemented with dextrose (0.4%), to reach a ∼ 5 × 10^5^ CFU/mL inoculum and distributed over a 96 well plate in triplicates. The ozone patch was applied on top of the plate for 6 h at room temperature. Aliquots of 10 μL were collected from each well every 1–2 h, mixed together to generate a representative population, serially diluted in PBS and each dilution was plated onto TSA plates and incubated overnight at 37°C. For staining, 100 μl of bacterial suspension was taken at 0–6 h. The harvested cells were stained with Live/DEAD *Bac*Light Bacterial Viability Kit (Thermo Fisher Scientific) according to manufacturer protocol. The dye solution (SYTO 9, propidium iodide) has excitation/emission wavelength of 485/498 and 535/617 nm, respectively. The stained bacterial cells were observed with an inverted fluorescence microscope (Olympus, Waltham, MA, United States) equipped with a camera under a 40× objective and 10× optical lens using a NIS-Elements D software. In this technique both live and dead cells are stained fluorescent green by SYTO 9 and dead or membrane compromised cells are re-stained red by propidium iodide, masking the initial green stain on the dead cells (Boulos et al., 1999; Dey et al., 2019).

In order to investigate the effect on the patch of long-term exposure to biofluid, an additional study was undertaken. This study tested the antimicrobial performance of the system through a pristine patch and a secondary patch pretreated with cell culture media as a simulation of biofuild. The patch contact study was conducted by inoculating *Pseudomonas aeruginsa* in 5% TSB overnight at 37°C. 50 μL of the solution was spotted onto cellulose filter paper, which was placed on petri dish and covered with Parafilm. The preconditioned patch was placed on top of a sponge soaked in Dulbecco’s Modified Eagle Media (DMEM), covered, and left overnight, while pristine patch samples were dry and unaltered. Experimental samples were exposed to ozone treatment (12V ozone input, 8V blower input) through a patch for 6 h, while control samples were left untreated. Once experimentation was complete, the filter paper samples were added to 1 mL of 0.05 vol% Tween20 and PBS and shaken for 15 min. The solution was then serially diluted and spotted on an agar plate as described above, and incubated overnight in 37°C. In each case, colony counts were collected for each spot, and averages were calculated along with the standard deviation.

### Cytotoxicity Tests

Human mammary stromal fibroblasts, HMS-32 cells (between passages 4 and 6) were cultured in Dulbecco modified Eagle medium (DMEM)/F12 medium (Invitrogen Inc., Carlsbad, CA, United States) supplemented with additives, insulin (250 ng/ml; Sigma-Aldrich), hydrocortisone (500 ug/ml; BD Biosciences, San Jose, CA, United States), sodium selenite (2.6 ng/mL; BD Biosciences), transferrin (10 ug/mL; Sigma-Aldrich), supplemented by transforming growth factor beta 1 (TGFβ1) (30 pg/ml; Thermo Fisher Scientific, Waltham, MA, United States), and fibroblast growth factor (FGF) (5 ng/ml (Thermo Fisher Scientific). Cells were seeded at a density of 5000 cells/cm^2^ for HMS32 on 18 mm glass cover slips placed in 12-well plates and were cultured at 37^*o*^C in a humidified environment (95%) with 5% carbon dioxide as described before (Chittiboyina et al., 2018). Cells were exposed to ozone via the patch on day 6 of culture for 6 h and cells were maintained for 6, 24, and 48 h post exposure along with respective untreated controls.

To test for the effect of ozone on cell survival and viability, immunostaining for apoptotic cells was done using antibody against Caspase-3 (Cell signaling technologies, Boston, MA, 1:400 dilution) was used. Cells fixed with 4% paraformaldehyde (Sigma-Aldrich) and permeabilized with 1% Triton were processed for immunostaining as described previously (Plachot and Lelièvre, 2004). Cytoplasmic actin cytoskeleton was stained with Alexaflour @480 conjugated phalloidin (Thermo Fisher Scientific; 1:40 dilution). Nuclei were counter-stained with DAPI (500 μg/ml). After mounting on antifade and sealing the coverslips, images were recorded using Q-capture image acquisition software linked to a BXI70 inverted fluorescence microscope (Olympus, Waltham, MA), with 20× objective (NA = 0.45). A total of three technical replicates per condition were evaluated with an average of 100 nuclei scored per replicate. As before, average values of the data were calculated along with standard deviation.

## Results and Discussion

### Patch Performance Assessment

**FIGURE 3:**
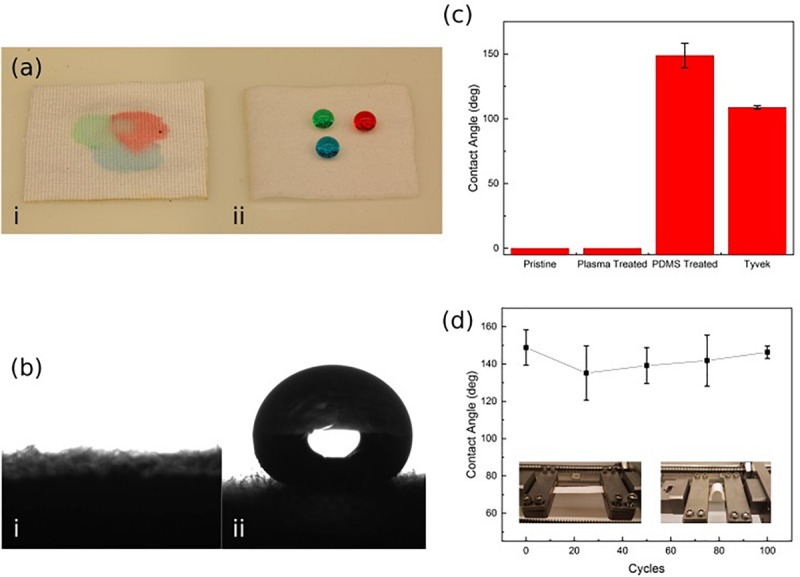
**(a,b)** Hydrophobicity of untreated (i) and PDMS treated (ii) Rayon-Spandex knit fabric sample. **(c)** Contact angle of patch surface compared at different steps in the treatment process and Tyvek. **(d)** Contact angle over repeated loading cycles. Data is the average of three samples, with error bars indicating standard deviation.

It was determined that the wound-touching surface of the patch must be hydrophobic to prevent the wicking action of the wound exudates, drawing the biofluid into the patch’s structure and filling the pores; thus, reducing the ability for the ozone gas to penetrate through the patch. The hydrophobicity of the patch (shown in Figures 3a,b) was evaluated by measuring the contact angle of water droplets on the Rayon-Spandex knit fabric. An initial measurement was taken of the patch before any treatment, after plasma treatment, and again after PDMS coating. As seen in Figure 3c, initial contact angle measurements for both the untreated patch and the plasma treated patch were 0°, meaning the droplet was completely absorbed into the material. For the PDMS treated fabric (PDMS-fabric), the contact angle between the patch and the droplet was measured to be between 135°–150° with super-hydrophobic water repelling properties. The PDMS-fabric showed a superior hydrophobicity as compared to the commercially available polymer film such as Tyvek (CA of 109°).

In order to verify that the PDMS-fabric would remain hydrophobic throughout the use, contact angle measurements were taken over a series of loading cycles. This loading simulated numerous movement cycles of the patch over its lifetime while attached to a user. To implement the loading, the PDMS treated patch was repeatedly exposed to a compressive bending force. In each cycle, the patch sample was bent with a radius of curvature equal to 13.5 mm. These bending cycles were applied in 25 cycle increments, and the contact angle measurement was taken after each set of 25 cycles, up through 100 cycles. Figure 3d shows the measured approximate contact angle remained constant throughout the testing cycles.

As shown by the initial hydrophobicity results, the PDMS patch successfully resisted the water droplets, while the two untreated samples did not (Figure 3ai,ii). The high level of hydrophobicity created by this approach is apparent when the PDMS patch contact angle results of 135°–150° are compared to those of Tyvek (109°), which is a commercially available hydrophobic, permeable polymer sheet. The developed patch shows a much higher resistance to the water due to the nature of the PDMS within the material, which allows it to exceed the standard set by Tyvek.

**FIGURE 4:**
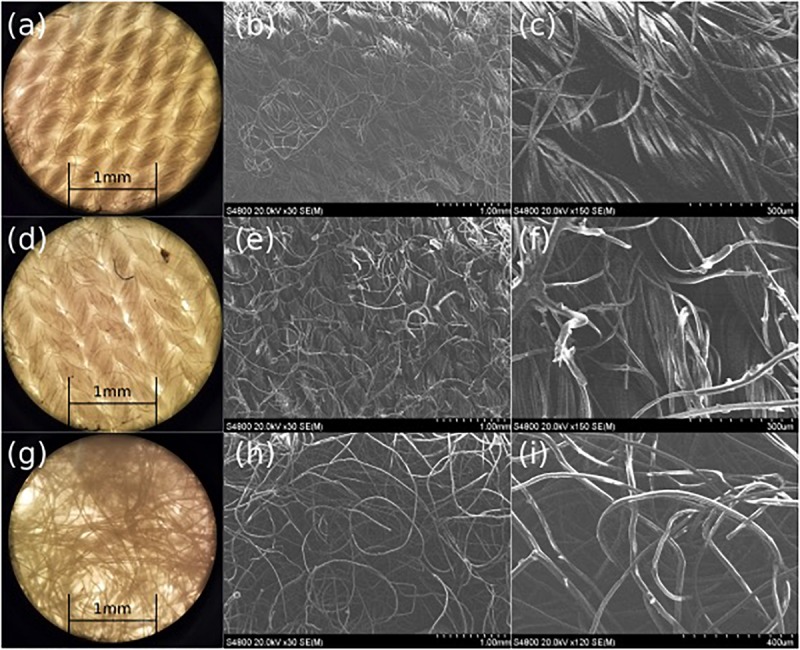
Microscope **(a)** and SEM **(b,c)** images of pre-treated Rayon-Spandex, PDMS treated Rayon-Spandex fabric (**d:** microscope, **e**,**f:** SEM) and intermediate flow dispersion layer (**g:** microscope, **h,i:** SEM).

The effects of PDMS treatment on the Rayon-Spandex microstructure and porosity were also investigated. We used optical and SEM microscopy to analyze the fiber thickness and pore size of the patch material before and after PDMS treatment (Figure 4). As can be seen, the PDMS treatment results in a coating of some of the fibers. It is this coating that provides the hydrophobicity observed in the previous section. The treatment doesn’t significantly alter the physical structure of the fibers and any change in the porosity due to the PDMS treatment is negligible. Microscopic analysis was also performed on the flow dispersion layer (Figure 4); the larger and randomly orientated fibers help distribute the ozone gas more uniformly throughout the patch. The dispersing layer has larger pore size distribution (0.003–0.02 mm^2^) as compared to the fabric layer (0.002–0.008 mm^2^), creating a pore “gradient” within the patch.

**FIGURE 5:**
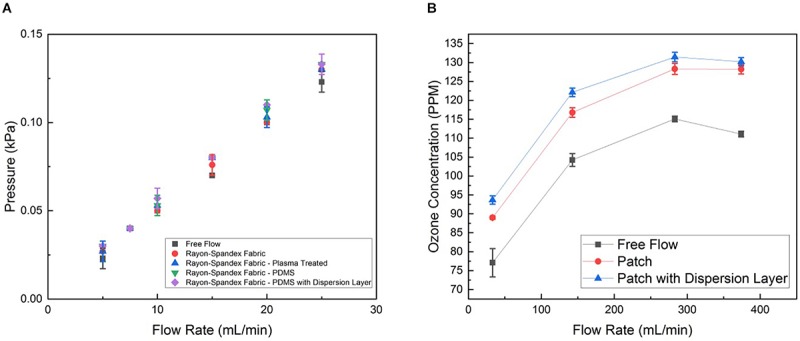
**(A)** Permeability characterization of the patch material before and after PDMS coating with and without the dispersion layer, represented by internal pressure from flow resistance measured at different flow rates generated by syringe pump. **(B)** Ozone generation of patch operated with and without dispersion layer at different flow rates generated by the micro-blower. Data is the average of three samples, with error bars indicating standard deviation.

Subsequently, we quantified the ozone flow resistance through the patch. To do this, the internal pressure of the flow was measured at a range of flow rates for the patch at different stages. This indicates how much the PDMS and intermediate dispersion layer impede the delivery of the ozone through the patch. Figure 5A shows the flow resistance characterization of the patch material before and after the PDMS coating with and without the intermediate flow dispersion layer. As can be seen, the PDMS coating has insignificant effect on the flow resistance, leading to no increase in the internal pressure at any flow rate. When the flow dispersion layer was added, there was no discernable increase in the flow resistance (the slope without the intermediate layer being 0.0052 kPa mL^–1^ min^–1^, whereas adding it changed the slope to 0.0053 kPa mL^–1^ min^–1^).

We also characterized the concentration of ozone delivered through the patch. The ozone concentration at the patch output (i.e., the surface touching the wound) was measured for four different inputs to the micro-blower, each with and without the intermediate flow dispersion layer in the patch and without any patch, while the output from the ozone generator was held at a constant 4 mg h^–1^ (Figure 5B). As expected, at low flow rates the micro-blower delivers lower ozone concentrations at the patch surface (∼ 90 ppm). Driving the micro-blower harder increases the ozone concentration up to 130 ppm after which the concentration starts to decreases. The free stream concentrations are lower than those using the patch, both with and without the dispersion layer by about 10%. This is likely due to how the ozone generation scheme interacts with the change in flow behavior due to the presence of the patch. Ozone is generated a constant mass rate, so the concentration delivered is a function of the flow rate. When the flow dispersion layer is added, the flow is slowed down and spread out. Thus, a slightly higher concentration is measured as the same amount of ozone is contained within a smaller volume of gas (a 1% increase).

**FIGURE 6:**
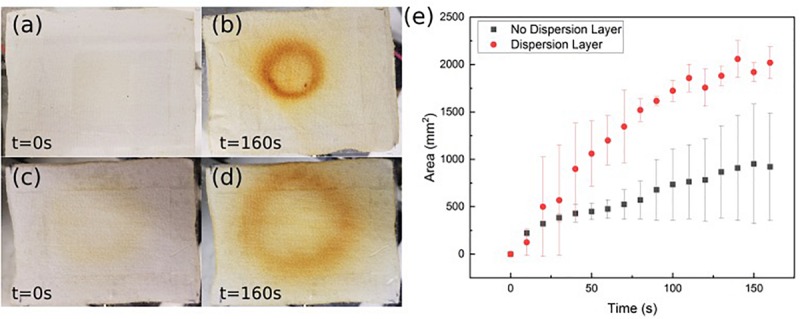
Ozone detection strip images without intermediate flow dispersion layer (**a**, before; **b**, after) and with intermediate flow dispersion layer (**c**, before; **d**, after). **(e)** Area measured by detection strip over time. Data is the average of three samples, with error bars indicating standard deviation.

To better characterize the ozone distribution at the patch surface, time dependent ozone concentration was mapped using an ozone sensitive test strip. The strip was placed on top of the patch while ozone was forced through and pictures were taken at regular intervals. Figures 6a–d shows the qualitative 2D distributed ozone generated by the patch with and without the intermediate dispersion layer. Figure 6e shows the effective ozone treated area over time with and without the dispersion layer. As can be seen in both the images and the graph, the intermediate flow dispersion layer increased the effective treatment area. There was a greater than 250% increase in the total effective treated area detected using the porous intermediate dispersion layer in the patch over 160 s.

**FIGURE 7:**
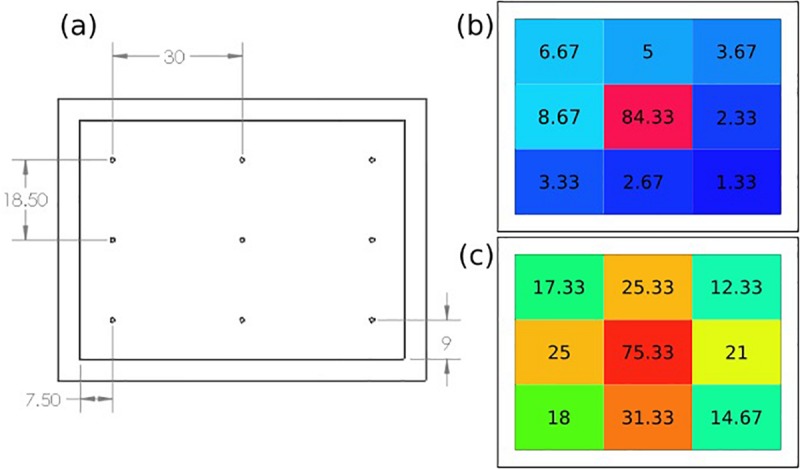
**(a)** Ozone distribution detection points on patch, results of recorded ozone concentration delivery map (ppm), **(b)** without, and **(c)** with intermediate flow dispersion layer. Each measurement the average of three data points at location.

The spatial dispersion of the ozone through the patch was also assessed by directly measuring the ozone output levels at nine select points across the patch as shown in Figure 7a. Figure 7b shows the recorded ozone concentration levels (ppm) at each patch location without the intermediate flow dispersion layer, and Figure 7c shows concentration values with the dispersion layer. It can be seen that greater concentrations of ozone reached the outer portions of the patch when the intermediate flow dispersion layer was included. This confirms the design provides greater dispersion of ozone across the treatment area.

### Antimicrobial and Cytotoxicity Assessments

**FIGURE 8:**
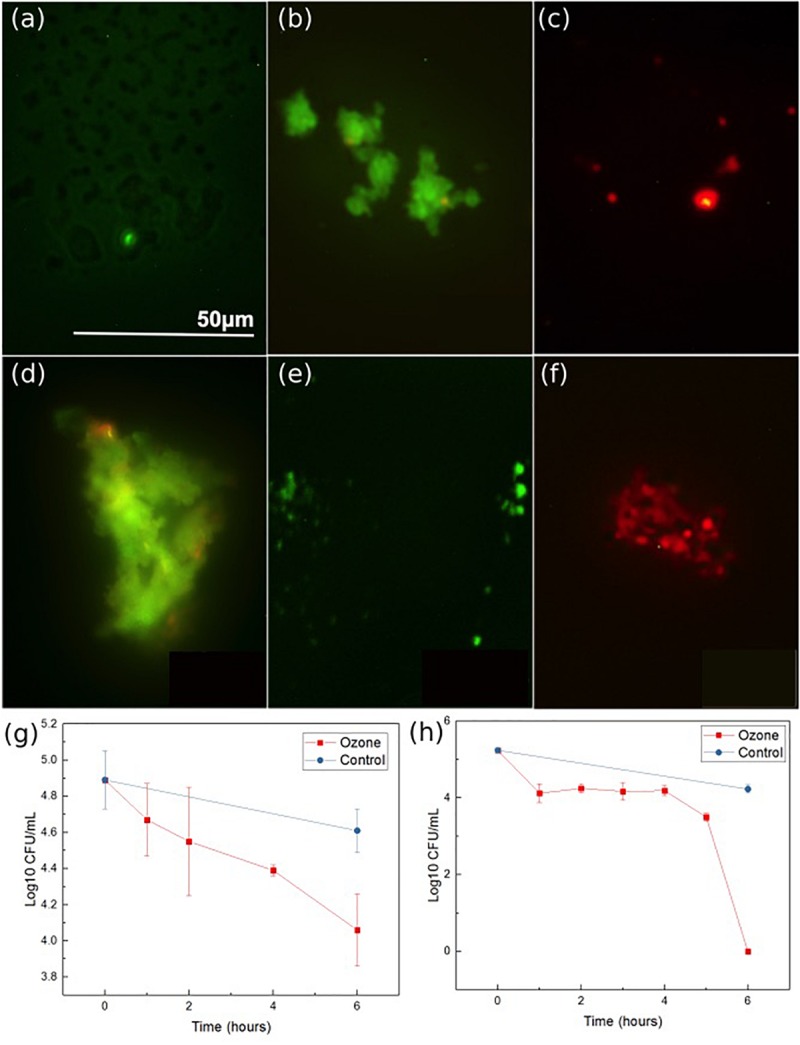
Ozone therapy results for *S. epidermidis*: stain imaging of **(a)** initial, **(b)** control at 6 h, **(c)** ozone treatment at 6 h, *P. aeruginosa:* stain imaging of **(d)**, initial **(e)** control at 6 h, and **(f)** ozone test at 6 h. *P. aeruginosa* **(g)** concentration graph during ozone treatment and *S. epidermidis* **(h)** concentration graph during ozone treatment. Data was collected from triplicate samples, with error bars indicating standard deviation.

The antibacterial properties of the developed ozone treatment patch were assessed by exposing multiple strains of antibiotic resistant bacteria (both Gram-negative and Gram-positive) to the ozone applied by the patch for 6 h, and measuring the living concentration every 1–2 h. Experiments using two common strains of antibiotic resistant bacteria common in skin infections, *S. epidermidis* NRS101 and *P. aeruginosa* ATCC 15442, indicated positive results. Over the course of 6 h of exposure, the Gram-positive bacteria *S. epidermidis* showed greater than 70% reduction from the starting inoculum (0.8 log_10_ reduction CFU/mL) (Figure 8g). Figures 8a–c are bacteria stain images of the samples before and after treatment, and a control. At first, all of the bacteria are stained green, and then any dead bacteria are re-stained with the red. These images show qualitatively that the bacteria before the treatment are living, and afterword are mostly dead. The results indicate that a single ozone treatment/exposure can be used to eliminate a substantial portion of the present bacteria. Additionally, results for the Gram-negative bacteria, *P. aeruginosa*, indicated even better results. When tested in the same conditions, the device was able to completely eradicate the initial 5 log CFU/mL concentration of *P. aeruginosa* within the 6 h of treatment, while the control population was again relatively unchanged (Figure 8h). Figures 8d–f show similar stain images for *P. aeruginosa.* The difference in the response between *S. epidermidis* and *P. aeruginosa* is likely due to the different structure of the outer cell walls. Despite this difference, both are shown to be oxidized by the ozone, leading to cellular collapse. The results of typical antibiotics on *P. aeruginosa* and *S. epidermidis* are already known. In both cases, there are antibiotics that are known to be effective [Colistin, Meropenem for *P. aeruginosa* (Hill et al., 2005) and rifampicin and vancomycin for *S. epidermidis* (Haque et al., 2009)], as well as those which are resisted [Penicillin and Tobramycin for *P. aeruginosa* (Hancock and Speert, 2000) and penicillin and oxacillin for *S. epidermidis* (Haque et al., 2009)]. As such, these results, for both Gram-positive and Gram-negative bacteria, indicate that ozone treatment can be an effective solution for antibiotic resistant bacteria strains.

**FIGURE 9:**
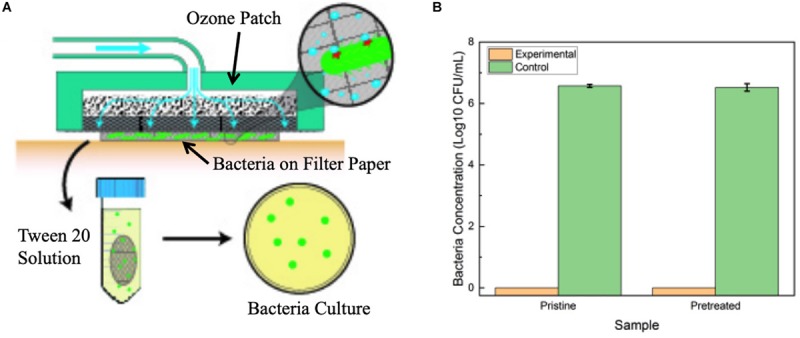
**(A)** Diagram of experimental setup for testing the effect of prolonged patch exposure to biofluid. **(B)** Antimicrobial results of pristine patch and pretreated patch in contact treatment of *P. aeruginosa* over 6 h. Error bars indicate standard deviation.

A secondary study was also conducted on P. aeruginosa to verify that prolonged contact to a wound environment would not change antimicrobial performance of the patch. Samples of *P. aeruginosa* were spotted onto filter paper for testing. First, treatment was applied through a pristine patch that was placed on top of the filter paper for the 6-h exposure time, and results were compared to a second filter paper sample that was exposed to no treatment. Afterward, the experiment was repeated, but utilizing a patch that had been preconditioned by exposure to cell culture media overnight to simulated prolonged contact with a wound site. Figure 9 reports the results of the tests. The treatment through a pristine and preconditioned patch both showed complete elimination of the *P. aeruginosa* after the 6 h of treatment. This verifies that long-term contact between the patch and a simulated wound environment did not inhibit ozone treatment.

**FIGURE 10:**
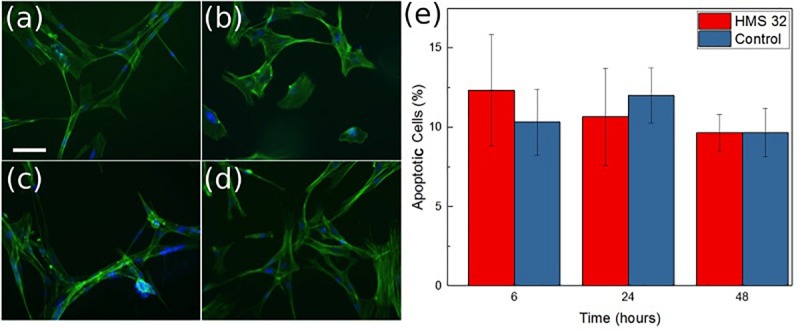
Images of normal mammary fibroblasts HMS-32 cells **(a)**, initial **(b)** after 6 h of ozone treatment, **(c)** 24 h after ozone treatment, and **(d)** 48 h after ozone treatment. Scale bar 10 μm. Graph showing percentage of apoptotic cells at each time in control and experimental group **(e)**. Data was collected from triplicate samples, with error bars indicating standard deviation.

The possibility for the use of ozone to treat antibiotic resistant infections is also dependent on the device not damaging the normal skin cells. Cytotoxic experimentation was conducted to determine how the ozone treatment would interact with human skin cells (Figures 10a–d). Contact effects of the patch were not directly included in the biocompatibility test because both Rayon-spandex fabric and PDMS have been shown to be biocompatible (ATEX, 0000; Gupta, 1998; Ionescu et al., 2012). Ozone treatment has been used to increase the healing process for chronic wounds, so there is good reason to believe a high level of biocompatibility exists. Figure 10e shows the number of viable cells observed after 6 h of ozone exposure, which was the similar duration of exposure used for bacteria. It also shows results 24 and 48 h after treatment to determine if ozone treatment caused any detrimental effect on the cells over time. It can be seen that there were no significant increase in cell apoptosis cells after 6, 24, and 48 h (6.5% over 48 h). Similar to the stain imaging done for the antimicrobial results, Figures 10a–d are stain images that show the health of the cells at each time point. In each cell, F-actin is stained green, and the nuclei are stained blue. These images support finding minimal cytotoxicity because there is no noticeable difference in the images over time. At the applied dosage, it can be concluded that ozone, as a treatment method, produces no negative effects on the healthy human cells it contacts.

The antimicrobial properties of ozone delivered through the patch seems to be effective in reducing or eliminating bacteria that often cause antibiotic resistant wound infections. The concentrations of ozone delivered topically (90–130 ppm), while still larger than the allowed EPA concentrations, are still confined to the wound area and much easier to contain or filter for safe application. Ozone therapy may work synergistically with traditional antibiotic treatments when used in a combination therapy. One of the mechanisms of bacterial resistance against antibiotics is development of changes in cell membrane and hence preventing entrance of antibiotic into the cell (Stapleton and Taylor, 2002). By oxidizing the outer layer of the bacteria, ozone could eliminate this barrier and allow the antibiotics to enter the bacterial cell in order to be effective. Finally, ozone exposure over a given time can accelerate the wound healing by inducing oxidative stress to the cells, stimulating the protective mechanisms of cells and organs, therefore, increasing the efficacy of endogenous oxygen free radicals’ scavenging properties as well as enhancing the Krebs cycle production of ATP (Filippi, 2001; Sen et al., 2002; Martínez-Sánchez et al., 2005; Lim et al., 2006; Valacchi et al., 2011; Wainstein et al., 2011; Zhang et al., 2014; Degli Agosti et al., 2016).

## Conclusion

Antibiotic resistant infections are a growing public health concern. A promising alternative to antibiotic therapy is utilizing the antimicrobial properties of topical ozone treatments. Developing a portable system designed to apply ozone to a targeted area will increase the options patients have in fighting infections that may otherwise be difficult to treat. In this work, we developed an ozone-releasing wound dressing consist of a disposable gas permeable hydrophobic patch with a reusable and portable ozone-generating unit. The patch incorporated a hydrophobic and highly ozone permeable outer layer and an inner dispersion layer for increased gas distribution uniformity. The antimicrobial effects of the system were tested against common antibiotic resistant strains of bacteria. The results indicated complete elimination of *P. aeruginosa* and significant reduction in the number of *S. epidermidis* colonies after 6 h of exposure. These tests also showed a high level of biocompatibility (low cytotoxicity) with human fibroblast cells during the same duration ozone treatment. The described patch is a promising tool in the management of chronic infected wounds.

## Data Availability Statement

The datasets generated for this study are available on request to the corresponding author.

## Author Contributions

AR was responsible for design, construction, and characterization of the ozone generation device and patch. AE conducted the microorganism experiments. VS performed the microorganism imaging. RS conducted the biocompatibility experimentation and imaging. MS, BZ, and RR provided the direction and oversight to the project.

## Conflict of Interest

The authors declare that the research was conducted in the absence of any commercial or financial relationships that could be construed as a potential conflict of interest.
