# Supplementary material for: Wearable and Flexible Ozone Generating System for Treatment of Infected Dermal Wounds
Source: Front Bioeng Biotechnol. 2020 May 19;8:458. doi: 10.3389/fbioe.2020.00458 (PMC7249782; doi:10.3389/fbioe.2020.00458)
Supplement: Supplementary file 1 [file Data_Sheet_1.PDF]

Cost analysis

Disposable Components	Unit Cost	Total Cost	Reference
PDMS	\$121.17/kg	\$2.4234	1
Rayon-Spandex Fabric	\$4/m^2	\$0.0019	2
Polyester Batting	\$3.60/m^2	\$0.0013	3
3M 300LSE Tape	\$130/m^2	\$0.0169	4
**Total cost of each disposable patch**		**\$2.44**	**This work**

Reusable portable Ozone delivery system	Unit Cost	Total Cost	Reference
Ozone Generator	\$40/ea	\$40	5
Microblower	\$12/ea	\$12	6
Microcontroller	\$13.39/ea	\$13.39	7
Tubing	\$0.0027/mm	\$1.37	8
**Total**		**\$66.76**	**This work**

Comparison to other treatment devices

Device	Cost	Portable	Reference
Ozone Generation System	\$66.76	Yes	**This work**
VAC Pump	\$295	Yes	9
Clinical Ozone System	\$1000/session	No	10
Hyperbaric Oxygen system	\$250/session	No	11

Comparison to other commercial patches

Product	Disinfection Method	Application Time	Cost	Reference
Ozone Patch	Ozone Delivery	6 hours	\$2.44	**This work**
Acticoat Patch	Silver	3 days	\$6.44/ea	12
DermaBlue Foam	Silver Sodium Zirconium Phosphate	Up to 3 days	\$115.2/ea	13
IodoFoam	Iodine	-	\$27.65/ea	14
SilvaKollagen Gel	Silver Oxide	-	\$75/tube (1.5oz)	15

Additional Figures

Absorption Study

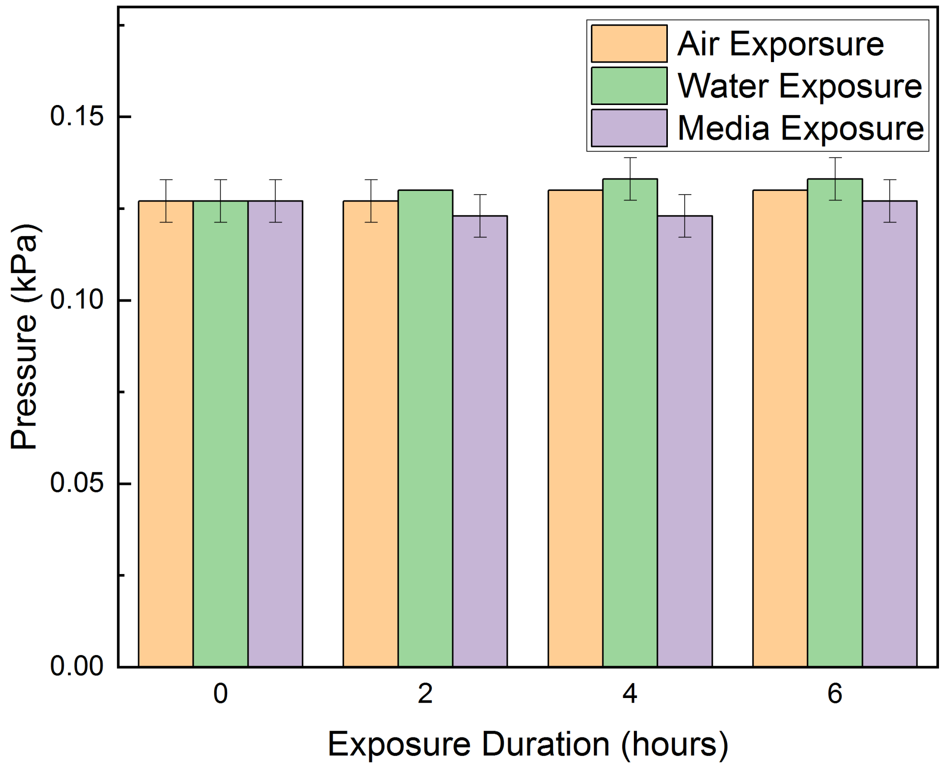

Figure 1S Permeability characterization of the PDMS-treated fabric over six hours of contact with deionized water, cell culture media, and air. Data is the average of three samples, with error bars indicating standard deviation.

A study was done to verify that the bulk hydrophobicity of the treated fabric would be sufficient to prevent prolonged contact with fluid from increasing flow resistance. Measurements of the internal flow pressure were taken as constant air flow was passed through PDMS treated fabric samples at a flow rate of 25mL/min after 0, 2, 4, and 6 hours of direct contact with different liquid environments. Water was chosen as the test fluid because it is the main component of biofluids, and would be even more susceptible to absorption due to its lower viscosity (0.6-0.75 mPa*s for water vs. 1.1-1.3mPa*s for plasma), and cell culture media was used as a simulation of biofluid. As seen in **Figure 1S**, the prolonged contact with the different fluids did not result in any noticeable change in flow resistance of the PDMS treated patch. This verifies the claims above that the hydrophobic nature throughout the bulk of the patch is sufficient in keeping pore from being clogged via absorption of a liquid. In each case, measurements were taken in triplicate.

References

[1] Dow Sylgard PDMS, https://www.ellsworth.com/products/by-market/consumer-products/encapsulants/silicone/dow-sylgard-182-silicone-encapsulant-clear-19.9-kg-kit, accessed: March 2020

[2] Rayon-Spandex Fabric, https://www.fabricwholesaledirect.com/products/ity-knit-fabric?variant=1244030071&gclid=CjwKCAiA-vLyBRBWEiwAzOkGVASaWJ6sItuRZo3Tl_iLOhhcMudVpmuhc572nh6bhj03b4xa3IlqixoChXAQAvD_BwE, accessed: March 2020

[3] 3M 300LSE Tape, https://www.amazon.com/Soles2dance-super-strong-double-sided-packaging-3M9474-08x12/dp/B00CCGK3GG/ref=pd_bxgy_3/135-5095665-9692106?_encoding=UTF8&pd_rd_i=B00CCGK31G&pd_rd_r=d564d17a-863c-40e2-bc0b-a6276df876a3&pd_rd_w=1az6D&pd_rd_wg=ou6Q1&pf_rd_p=fd08095f-55ff-4a15-9b49-4a1a719225a9&pf_rd_r=RNMTSH62JHK8MXDDTQ4X&refRID=RNMTSH62JHK8MXDDTQ4X&th=1, accessed: March 2020

[4] Polyester Batting, https://www.joann.com/4oz-x-10-yd-batting/7066947.html, accessed: March 2020

[5] Murata MHM500-00A Ozone Generator, https://www.mouser.com/ProductDetail/Murata-Electronics/MHM500-00A?qs=%2Fha2pyFaduhGwQoxJATVGYDiVsMS3CriV88oqAcmxtI3DK7fBdnE8g%3D%3D, accessed: March 2020

[6] Murata MZB1001T02 Microblower, https://www.mouser.com/ProductDetail/Murata-Electronics/MZB1001T02?qs=yQ3iditm8N7IjFJvzD7ZFg%3D%3D, accessed: March 2020

[7] Arduino Micro Microcontroller, https://www.mouser.com/ProductDetail/Arduino/ABX00033?qs=sGAEpiMZZMve4%2FbfQkoj%252BAOOX4xr%252BR35xxX63G3%252BecQ%3D, accessed: March 2020

[8] Silicone Tubing, https://www.amazon.com/Silicone-Tubing-Foot-Piece-D/dp/B00ECFHYHQ/ref=asc_df_B00ECFHYHQ/?tag=hyprod-20&linkCode=df0&hvadid=312131715276&hvpos=&hvnetw=g&hvrand=14318737768216267799&hvpone=&hvptwo=&hvqmt=&hvdev=c&hvdvcmdl=&hvlocint=&hvlocphy=9016722&hvtargid=pla-669139988874&psc=1, accessed: March 2020

[9] Smith & Nephew 66800955 PICO Portable VAC System, https://www.medicalmega.com/medical/smith-nephew/66800955.html?gclid=CjwKCAiA-vLyBRBWEiwAzOkGVEJR670TynWNmidms43mTlgSSLs6_INIsWztJrTD0yXGIajbZkHP8BoCe3wQAvD_BwE, accessed: March 2020

[10] Ten Pass Commercial Ozone Treatment, https://www.prohealth.com/library/ten-pass-ozone-the-latest-and-greatest-tool-for-lyme-disease-42886, accessed: March 2020

[11] Hyperbaric Oxygen Treatment, https://www.healingdives.com/faqs/typical-cost-treatment, accessed: March 2020

[12] Acticoat Dressing, https://www.adwdiabetes.com/product/1722/smith-nephew-acticoat-dressing?utm_source=google&utm_medium=cpc&utm_campaign=shopping&gclid=CjwKCAiA-vLyBRBWEiwAzOkGVAmo2gGpTURhtgvAvCeAbJ7ppWSBHEI0viI8TUy_xsb3ijkocz6dXRoCG00QAvD_BwE, accessed: March 2020

[13] DermaBlue Dressing, https://www.cleanitsupply.com/p-148083/l-cs7pp/dermablue-foam-foam-dressing-with-silver-polyurethane-foam-5-bx-1053960_bx.aspx?gclid=CjwKCAiA-vLyBRBWEiwAzOkGVFfMj2UD2kxxMeVXQH4m2kbuaaOq8E8s621mu-ChRZqCaxa1kxB0EhoCpQgQAvD_BwE, accessed: March 2020
[14] Iodofoam Dressing, https://www.healthproductsforyou.com/p-iodofoam-iodophor-foam-wound-dressing.html, accessed: March 2020
[15] Silvakollagen Gel, https://www.healthproductsforyou.com/p-dermarite-silvakollagen-gel.html, accessed: March 2020
